# Taguchi grey relational optimization of sol–gel derived hydroxyapatite from a novel mix of two natural biowastes for biomedical applications

**DOI:** 10.1038/s41598-022-22888-5

**Published:** 2022-10-26

**Authors:** Obinna Anayo Osuchukwu, Abdu Salihi, Ibrahim Abdullahi, David Olubiyi Obada

**Affiliations:** 1 Department of Mechanical Engineering, Bayero University, Kano, 700241 Kano State Nigeria; 2 Department of Mechanical Engineering, Ahmadu Bello University, Zaria, Samaru Zaria, 810212 Kaduna State Nigeria; 3 Africa Centre of Excellence on New Pedagogies in Engineering Education, Ahmadu Bello University, Zaria, Samaru Zaria, 810212 Kaduna State Nigeria; 4 Multifunctional Materials Laboratory, Shell Chair Office in Mechanical Engineering, Ahmadu Bello University, Zaria, Samaru Zaria, 810212 Kaduna State Nigeria

**Keywords:** Engineering, Materials science, Mathematics and computing

## Abstract

The comparative study of natural hydroxyapatite (NHAp) from bovine (B) and catfish (C) bones using different fabrication parameters has been extensively researched through traditional investigation. However, the quantitative effect optimization of a novel mix proportion of hydroxyapatite from these bones, and fabrication parameters have not been examined. Hence, this study presents the effect of the powder mixture, compaction pressure, and sintering temperature (as production parameters) on the experimental mechanical properties of naturally derived HAp. The bovine bone and catfish bone biowastes were used in mixed proportions to produce hydroxyapatite via the sol–gel synthesis protocol. The powders were calcined separately at 900 °C to convert the deproteinized biowaste. Next, the powders were combined chemically (sol–gel) in the appropriate ratios (i.e. 45 g of B: 15 g of C (B75/C25), 30 g of B: 30 g of C (B50/C50), and 15 g of B; 45 g of C (B25/C75)). Taguchi design supported by grey relational analysis was employed with an L9 orthogonal array. The Minitab 16 software was employed to analyze the Taguchi design. The result revealed an inconsistency in the powder mixture as the optimum state for individual mechanical properties, but the grey relational analysis (GRA) showed better mechanical properties with a powder mix of B50/C50, 500 Pa compaction pressure, and 900 °C sintering temperature. The obtained result further showed that the novel mix of these powders is a good and promising material for high-strength biomedical applications, having a contribution of 97.79% on hardness and 94.39% on compressive strength of HAp. The obtained experimental grey relational grade of 0.7958 is within the 95% confidence interval, according to confirmation analysis (CA). The optimum powder parameter was examined using X-ray diffraction (XRD), and its structure, size, and elemental makeup were examined using scanning electron microscopy (SEM) and energy dispersive spectroscopy (EDS) analysis. The sample had a higher degree of crystallinity and mean crystallite size of 80.42% and 27.3 nm, respectively. The SEM images showed big, gritty grains that are not tightly packed.

## Introduction

Hydroxyapatite (HAp) is a biocompatible calcium phosphate (Ca/P) that has a wide range of uses in both industry and medicine. The primary justifications for adopting hydroxyapatite in bone replacement and tissue engineering are its bioactive and osteoconductive capabilities as well as its similarity to a bone mineral component^1,2^. With increased mechanical properties, HAp is a feasible material for drug administration, bone defect fillers, bone cement, and other biomedical engineering applications in dentistry and orthopedics^3^. Also, it is generally known in tissue engineering that HAp is the most stable form of calcium phosphate and that it is one of the materials utilized the most frequently in bone regeneration, mostly due to its affinity for bone tissues. Recent works contain HAp that was produced using a variety of processing techniques and both natural and synthetic source^4–10^. It has been widely shown that HAp shares chemical components with the inorganic component in animal bones, which has led to its desired biocompatibility qualities, such as harmlessness and a lack of inflammatory effects^4,11,12^.

Some scientists have been engaged in the development of HAp from natural sources. Bovine bones^5,8,11^, fish bones^13–15^, porcine bones^8,16^, eggshells^10,17^, and seashells^18,19^ are a few of the natural sources now being examined. From their results, some of these precursors have their weaknesses, also their availability and ease of processing are other challenges. Sol–gel process, chemical precipitation, hydrothermal reaction, and solid-state reaction are a few of the methods that can be utilized to manufacture hydroxyapatite powder^3^. The macrostructure of the HAp extracted from corals is similar to that of human cancellous bone. Through meticulous procedures, this is possible. Due to its poor mechanical strength and potential failure to promote the dimensional reduction of defective areas before bone regeneration, the HAp produced by hydrothermal treatment of corals is limited in its therapeutic applications^20^. The HAp made from algae similarly resembles biological apatite found in bone in several ways. This is made by heating calcium carbonate from calcareous red algae to a temperature of roughly 700 °C. The porosity of the algae is preserved by this technique, but with low mechanical strength^21^. The HAp from bone sources using heat treatment preserves the typical bone morphological structure with 70% porosity and enables its use as a bone substitute product. Heat treatment is an effective alternative for acquiring deproteinized bone tissue^22,23^. When bone is heated at a high temperature, it turns into high crystallinity, organic-free HAp. The heat treatment also has the benefit of lowering the possibility of biological contamination and implant rejection while enhancing some mechanical qualities in the case of additional products made from animal bone^24^.

According to Sobczak et al.^25^, the method for creating HAp powders from waste is intriguing because of its economic worth and environmental benefits. They also noted that the HAp is resistant to being readily overwritten by human tissues because of its inherent resemblance to human bone. For the research of hydroxyapatite scaffolds for tissue engineering, Pan et al.^26^ improved and assessed HAp synthesized from catfish bones to achieve ideal handling situations and practical teaching ways. To create a porous scaffold utilizing the space holder method, Gross et al.^27^ combined titanium oxide (TiO2) nanoparticles with nanoclay (NC) made with NaCl microparticles. According to their findings, the scaffold should also have an organized permeable structure in addition to its large porosity (> 80%) to promote better cell seeding and tissue growth. Aghdam et al. used the space holder (SH) approach to create HAp scaffold (6 mm × 10 mm) using sodium bicarbonate (NaHCO_3_) as a porous agent. For use as a bone substitute, HAp was reinforced with 0, 5, 10, and 15 wt% alumina nanoparticles (ALN: 40–80 nm)^28^. Using the sol–gel technique, Obada et al.^29^ reinforced hydroxyapatite from animal bone with 15 wt% of kaolin (K-HAp). The pellets were formed under a pressure of 1 MPa in preparation for compressive and hardness tests. At 1100 °C, the K-HAp matrix produced compressive strength of 7.66 MPa as opposed to 0.89 MPa for the unreinforced HAp matrix. Uskoković et al.^30^ doped HAp with germanium to produce a new apatite and the germanates substituted the phosphates in stoichiometric HAp. Their result showed that the lattice expanded in parallel to the basal plane and along the screw axis of the calcium ion hexagons.

The Taguchi methodology is an optimization technique that directs a small number of experiments to simultaneously examine the noise of various constraints. This technique aids in selecting the most effective set of control constraints to guarantee the procedure is noise-resistant. The most popular Design of Experiment (DOE) is the Taguchi method, which employs many orthogonal arrays to compare and analyze various strata of each control characteristic^31^. The many acts and properties of hydroxyapatite can be changed using the Taguchi Grey Relational Analysis (GRA) design. When one of the many DOE study instruments has incomplete or ambiguous data, one of the primary strategic methods used is Grey Relational Analysis (GRA)^32^. The GRA converts complex reactions into a single response that optimization algorithms can predict (Minitab)^33^. Abifarin^34^ maximized the mechanical characteristics of hydroxyapatite (HAp) made from cow bones using the Taguchi GRA method. Bovine femur bone was employed by Ahmed et al.^35^ to manufacture HAp. Regression analysis was then applied to improve the mechanical and physical properties. The L16 orthogonal array, Taguchi, and ANOVA analysis methods were utilized by Googerdchian et al.^36^ to identify the best parameter mixes with the fewest possible trials. For the treatment of osteomyelitis, Kumar et al.^37^ created and enhanced hydroxyapatite-ofloxacin implants. An easy method of optimization (Box Benhken) was employed by Akpan et al.^38^ to prepare and improve the mechanical properties of HAp from fish bones.

To extract HAp from bovine and catfish bones, various techniques such as heat decomposition, calcination, or its combination with one or more additional techniques have been used^21,39^. The bones are heated up to 1400 °C in the furnace during the calcination process to eliminate some organic elements such as proteins, lipids, fat, pathogens, etc.^21^. These techniques resulted in the creation of HAp powder with various morphologies, stoichiometric compositions, grain sizes, and crystallinity levels^25^. In the previous works^13,15^, the authors studied the synthesis, characterization, and experimental data of natural hydroxyapatite produced from a new blend of animal bones. The bovine and catfish bones were used as their apatite is similar to human bone. This new HAp was produced to improve its mechanical properties.

According to the brief open literature study stated above, HAp has been produced from bovine and catfish bones, demonstrating the reliability of these sources as natural ones. It also shows that the Taguchi method of optimization can produce the best optimum parameters for enhancing the mechanical properties of NHAp. To the best of the authors' knowledge, however, none of the literature mentioned the optimization of naturally occurring HAp produced from animal matrix found in bovine and catfish bones using a sol–gel protocol. These bones are used because they are readily available and hence reduce the cost of producing HAp, which is essential in tissue engineering. In the present study, the Taguchi Grey Relational Analysis was employed to establish the optimum production parameters (powder ratio, sintering temperature, and compaction load) for the robust production of a novel mix of naturally derived HAp from biowastes. The bovine bones (B) came from an abattoir in the Nigerian city of Zaria, while the bones for the catfish bones (C) came from nearby eateries. After being deproteinized in an oven, the bones were independently processed. Following heat treatment, the two sources' generated powders were weighed and combined with a spatula in various ratios, yielding 100 g. (scale-down measures were applied). The as-mixed powders were then further homogenized using the sol–gel method in the designated ratios (B75/C25, B50/C50, and B25/C75). The as-produced HAp scaffolds (45:15 g (B75/C25), 15:45 g (B25/C75), and 30:30 g (B50/C50) were sintered at 700, 800, and 900 °C for 2 h with compaction loads of 300, 400, and 500 Pa. The scaffolds were subjected to hardness and compressive testing to capture the data required for the Taguchi design and grey relational analysis (TDGRA). The S/N ratio is used as a performance characteristic (PC) in the Taguchi technique to measure the process's strength and measure the degree of deviations from expected norms. The Grey Relational Generation was used to standardize the unique location arrangements to the equivalent array of 0 and 1. The correctness of the created model was assessed and compared using the Grey Relational Coefficients of determination and Grades. Additionally, hypothesis testing was done to determine the model's goodness of fit.

### An overview of Taguchi grey relational analysis

The Taguchi methodology is an optimization technique that directs a small number of experiments to simultaneously examine the noise of various constraints^40^. This technique aids in selecting the most effective set of control constraints to guarantee the procedure is noise-resistant. The most popular DOE is the Taguchi method, which employs many orthogonal arrays to compare and analyze various strata of each control characteristic^31^. Researchers have confirmed that it is the best way to get standardized items at effective costs^41–43^. More research is needed on the Taguchi approach^44^ for managing a variety of performance parameters. Using Minitab 12, GRA simplifies complicated replies into a single response that optimization algorithms can forecast^33^. To achieve this, the following steps are employed:

#### Experimental design

This includes the input parameters and their corresponding levels. Next is the Taguchi orthogonal array (OA) with the number of runs for the trial assessment.

#### Signal–noise (S/N) ratios (R) in the Taguchi design technique

The Taguchi approach can utilize by the OA to reduce variance and optimize process parameters. The S/N ratio is used as a performance characteristic (PC) in the Taguchi technique to measure the process's strength and quantify the degree of deviation from expected models^45^. A logarithmic function is produced by assessing the percentage of signal (mean) to noise (standard deviation) to estimate the S/N^46^. Higher S/N is suitable for reducing noise and the effects of overwhelming elements, resulting in improved HAp (product) quality^47^. When a higher hardness and compressive strength are expected^48^, the larger-the-better S/N type Eq. (1) can be adopted.

1 $$\left( \frac{S}{N} \right) = - 10\log_{10} \left( {\frac{1}{n} \mathop \sum \limits_{i = 1}^{n} \frac{1}{{a_{i}^{2} }}} \right)$$

The response rate of the ith trial in the OA for an experimental combination is a_i_, where n is the number of trials.

#### Multi-mechanical characteristics optimization with grey relational analysis

The Taguchi DOE method is suitable for a single mechanical feature. However, multi-mechanical characteristic optimization with Grey Relational Analysis (GRA) is the ideal approach when there are two or more mechanical qualities with distinct excellent characteristics. If it appears that the fixed data is unequal, the grey analysis might be applied to the data^49^.

#### The grey relational generation

Before being converted into a grey relational design, any values connected to a specific technique must be put to the test. This tactic is known as the grey relational generation^50,51^. The elements' roles are disregarded once the regular data and location arrangement is significantly more extensive. When the targets and the order of the components are altered, Grey’s Relational Analysis could potentially produce inaccurate findings. The unique location arrangements are then standardized to the comparable array of 0 and 1 using data pre-developing^51^. Larger-is-better and slighter-is-better are two superior qualities that can be used to categorize the desire for value conversion via GRA and the contribution of converted orders. To standardize the ranking for the slighter, the better features, apply Eq. (2):

2 $$X_{i} \left( K \right) = \frac{{maxy_{i} \left( k \right) - y_{i} \left( k \right)}}{{maxy_{i} \left( k \right) - miny_{i} \left( k \right)}}$$

where x_i_(k) denotes the normalized figure for the number of trials and y_i_(k) is the preliminary order of the average responses.

#### The grey relational coefficient and grade

After the order has been standardized, the deviation sequence of the location order is calculated using Eq. (3)^48,52^.

3 $$\Delta_{{{\varvec{oi}}}} \left( {\varvec{k}} \right)\user2{ } = \user2{ x}_{{\user2{o }}} \left( {\varvec{k}} \right)\user2{ } - \user2{ x}_{{\user2{i }}} \left( {\varvec{k}} \right)\user2{ }$$

Given: $\Delta_{oi} \left( k \right) = deviation sequence$; $x_{o } \left( k \right)$ = Location sequence and $x_{i } \left( k \right) =$ Comparability sequence. The next step is the computation of the grey relational coefficient (GRC) via Eq. (4)^48^:

4 $$\varepsilon_{i} \left( K \right) = \frac{{\Delta_{min} + \zeta \Delta_{max} }}{{\Delta_{oi} \left( k \right) + \zeta \Delta_{max} }}$$

where, ξ_i_(k) indicates the GRC of distinct response variable quantity estimated as a role of ∆min and ∆max, the smallest and biggest deviations of the individual response variable (RV). The unique coefficient represented by ζ, defined in the series ζ ∈ [0, 1], is usually established at 0.5 to assign alike bulks to each parameter. Just as given in Eq. (5), the grey relational grade is then calculated by averaging each RV's GRC.

5 $$\gamma_{i} = \frac{1}{n} \mathop \sum \limits_{i = 1}^{n} \varepsilon_{i} \left( k \right)$$

Given that γ_i_ signifies the worth of GRG gotten for the ith trial, n is the total sum of presentation features. After the optimum level of the factors is evaluated via GRG, the last stage is to envisage and confirm the eminence characteristics via Eq. (6)^34,53^:

6 $$\gamma_{predicted} = \gamma_{m} + \mathop \sum \limits_{i = 1}^{p} \gamma_{o} - \gamma_{m }$$

where γ_o_ signifies the highest average GRG at the optimum level of factors, and γ_m_ signifies the mean GRG. The quantity p designates the number of factors that affect the RVs.

#### Analysis of variance (ANOVA)

Which variable level balance has the greatest influence on the overall demonstration characteristics is determined using the ANOVA. This can be accomplished by first reducing the overall inaccuracy of the grey relational grade, which is calculated as the sum of the squared deviations from the absolute mean of the grey relational grade, and then dissecting contributions by individual factor and mistake. Start by using Eq. (7) to determine the precise number of squared deviations (SST) from the overall grey relational grade mean^34^.

7 $$SS_{T} = \mathop \sum \limits_{j}^{p} \left( {\gamma_{j} - \gamma_{m} } \right)^{2}$$

The mean of the GRG for the jth trial is j, and the number of trials in the orthogonal array is p. Similarly, the Fisher,^28^ F test can be used to figure out which factor significantly balances the presentation features. The shift in the factor combination usually marks the presenting aspects when the F rate is large^34,54^.

### Confirmation test

The T-test compares a group's means. Confidence interval (CI) is a study of how close an experimental outcome is to the expected outcome, which may be calculated using Eq. (8)^52,53^:

8 $$CI = \sqrt {F_{\alpha } \left( {1,f_{e} } \right)V_{e} \left[ {\frac{1}{{n_{eff} }} + \frac{1}{X}} \right]}$$

*F*_∝_(1, *fe*) = F ratio essential for α; α = risk; *f*_*e*_ = DOF of error; V_e_ = variance of error; η_*eff*_ = effective number of repetitions, which is the Eq. (9) below:

9 $$n_{eff} = \frac{S}{{1 + \left( {Total \,DOF\, of\, control\, factors} \right)}}$$

X = number of repetitions for trial confirmation; S = Sum of trials.

## Materials and methods

### Sample preparation

Raw bone samples of bovine (B) and catfish (C) were obtained from a local abattoir and a nearby eateries respectively, both in Kaduna state, Nigeria. The fish bones were first washed in hot water before being rinsed under running water. As described by Obada et al.^55^, raw bovine (B) and catfish (C) bones were collected and meticulously cleansed with a considerable volume of running water to remove any debris. Before calcination, the bones were dehydrated and decomposed in a carbonizing oven for one (1) hour. The carbonized bones were calcined at 900 °C for two (2) hours under ambient circumstances in an electric furnace with a 5 °C/min ramp and two (2) hours of holding time, then allowed to cool in the furnace. Before solution gelation (Sol–gel) treatment, the calcined samples were crushed with a metallic mortar and pestle and sieved through a 100 µm sieve to obtain a fine powder. The powders were prepared separately before the sol–gel mixture.

Next, 45 g of B powder was weighed and poured into a beaker, then mixed with 150 ml of distilled water and strongly swirled on a hot plate with a magnetic stirrer for one (1) hour, followed by 15 g of C powder for another one (1) hour of vigorous swirling. The hot plate was heated to 50 °C for 30 min before being elevated to 100 °C to generate a gel solution and the sample was tagged B75/C25. The step was repeated for 45 g of C powder with 15 g of B powder and also for 30 g of B powder and 30 g of C powder and the samples were tagged B25/C75 and B50/C50 respectively. The resultant gel solutions were dried in the oven before being crushed to a fine nano-HAp with a ceramic mortar and pestle. The compaction pressure (CP) of 300 Pa, 400 Pa, and 500 Pa was used to pelletize the samples (25 mm × 25 mm) to improve the mechanical properties due to decreased stress during HAp powder compaction^56^. To sinter the pelletized samples, temperatures of 700 °C, 800 °C, and 900 °C were chosen.

### Mechanical examination

An MHV10002 micro-hardness tester was used to determine the micro-hardness (HV) of the sintered scaffolds using the Vickers indentation. A 300 g applied load was placed on the scaffolds for a dwell time of 10 s. Each scaffold had a total of 5 indentations, yielding 5 accurate hardness readings. A 5 kN load cell-equipped universal testing machine (UTM) was used to assess the scaffolds' compressive strength. For accuracy, 5 scaffolds were examined for each circumstance.

### Characterization of the optimum sample

The X-ray diffraction (XRD) patterns of the produced powders were collected on a Rigaku Miniflex diffractometer with a copper tube ($\lambda$ = 1.5418 A) at a voltage of 40 kV and a current of 30 mA in the range between 20° ≤ 2*θ* ≤ 80°, and the crystallinity and crystallite sizes were computed with expressions as adopted by^57^. Scanning electron microscopy (SEM) operated at 15 kV was used to analyze the morphology of the optimum sample (Phenom ProX Desktop) equipped with Electron dispersive X-ray analysis (EDX) for elemental mapping. Low magnifications of 300 × and 500 × to analyze each sample. The porosity, apparent density, crystallinity, and crystallite size of the optimum production parameters were calculated using the formulae used by Obada et al.^55^.

### Design of experiment

**Figure 1:**
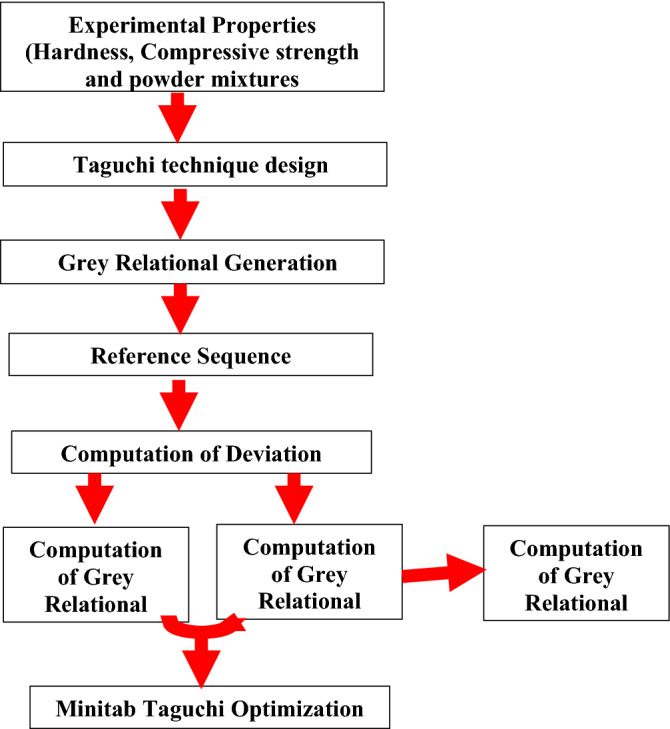
The Optimization steps.

The factors and their relative levels were developed using the Taguchi design technique and are displayed in Table 1 based on the design considerations. L9 was the appropriate orthogonal array to use, according to Minitab 16 software, and it is shown in Table 2. On Minitab, the Taguchi method was used to examine the matching experimental hardness, compressive strength, and resulting grey relational grade. According to Abifarin et al. and Awodi et al.^33,34^, the procedures for creating the final grey relational grade for the experimental properties are shown in Sect. 2.5. Using Minitab 16, all of the plotted graphs were created. The summary of the Taguchi-Grey relational optimization analysis is shown in Fig. 1.

**Table 1:** Processing parameters and their levels.

Processing parameters	Sintering temperature (ST) (°C)	Compaction pressure (CP) (Pa)	Powder mixture (PM) (% wt)
Level 1	700	300	B75/C25
Level 2	800	400	B25/C75
Level 3	900	500	B50/C50

**Table 2:** Experimental design strategy.

Experimental runs	Sintering temperature (°C)	Compaction pressure (Pa)	Powder mixture (% wt)
1	700	300	B75/C25
2	700	400	B50/C50
3	700	500	B25/C75
4	800	300	B50/C50
5	800	400	B25/C75
6	800	500	B75/C25
7	900	300	B25/C75
8	900	400	B75/C25
9	900	500	B50/C50

### Grey relational analysis

**Figure 2:**
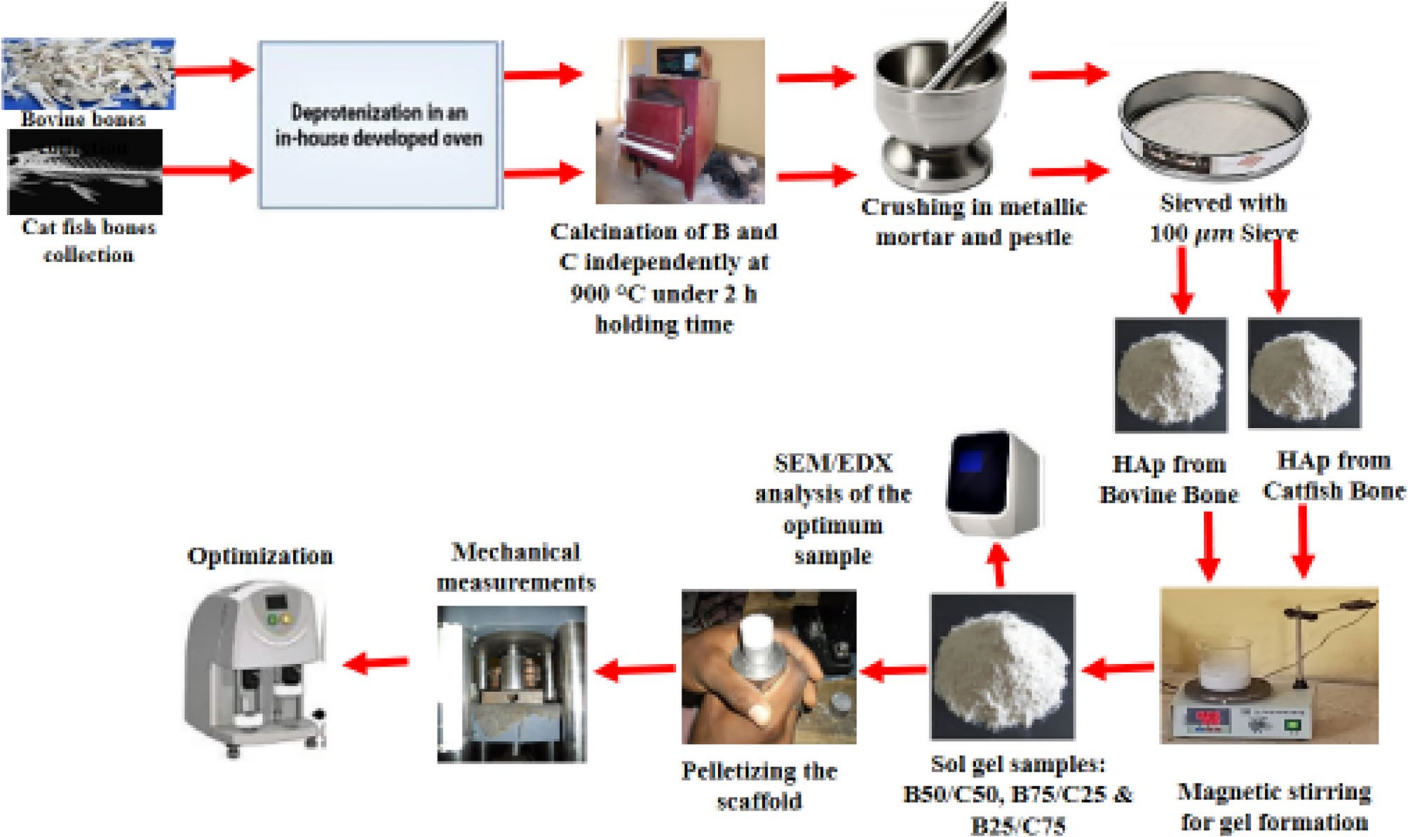
Synthesis, optimization, and characterization processes.

The efficiency of the Taguchi method for optimization can be increased by incorporating GRA ^58^. Experimental hardness and compressive strength cannot be averaged directly, hence grey relational analysis was used to address this problem ^32,59^. First, employing the larger-is-better principle Eq. (1), sub-Sect. Signal-Noise (S/N) ratios (R) in the Taguchi design technique), the values for hardness and compressive strength were transformed to grey relational generation (normalizing the sequence). Due to the desire for high compressive strength and hardness, the larger-the-better approach was used. After sequence normalization, Eq. (2) (Sub-Sect. The grey relational generation) was used to calculate the deviation sequence of the reference sequence. Equation 3 was then used to create the grey relational coefficient, and Eq. (4) was used to average the resulting hardness and compressive strength grey relational coefficients to create the grey relational grade (GRG) (see Sub-Sect. Analysis of Variance (ANOVA) for Eqs. 3 and 4). Figure 2 displays a schematic of the synthesis, optimization, and characterization processes.

## Results and discussions

### Taguchi orthogonal array (OA) of the mechanical characteristics result

Tables 3 and 4 display the OA of the mechanical properties and corresponding Taguchi S/N ratios of the treated HAp under stated conditions. Minitab 16 was used for the S/N ratios calculation and the data analysis.

**Table 3:** The highlights of the hardness experimental result (HS).

Number of runs	Hardness 1	Hardness 2	Hardness 3	Hardness 4	Hardness 5	Mean	S/N Ratios
1	31.7	43.5	50.7	51.7	49.0	31.7	30.0212
2	31.7	43.3	48.0	51.7	49.0	31.8	30.0212
3	31.8	43.5	50.1	51.8	49.1	31.8	30.0485
4	31.8	37.1	47.6	56.8	44.2	31.8	30.0485
5	32.0	37.1	46.7	56.7	44.2	32.0	30.1030
6	32.4	37.5	44.0	56.8	44.2	32.4	30.2109
7	34.0	41.2	45.0	55.7	50.0	34.0	30.6296
8	34.1	41.2	45.0	55.9	51.9	34.1	30.6551
9	34.0	41.0	45.1	55.9	51.7	34.0	30.6296

Hardness 1–5 = Number scaffolds used for Hardness tests.

**Table 4:** The highlights of the compressive trial result (CS).

Number of runs	Compressive strength 1	Compressive strength 2	Compressive strength 3	Compressive strength 4	Compressive strength 5	Mean	S/N ratios
1	600	500	900	900	600	600	55.5630
2	650	500	900	900	650	650	55.5630
3	650	200	900	500	1100	650	55.5630
4	650	400	1000	600	700	650	55.5630
5	700	400	1000	500	700	700	56.9020
6	700	500	900	500	600	700	56.9020
7	700	500	750	400	700	700	59.0849
8	750	800	750	500	800	750	59.0849
9	900	800	900	400	800	900	59.0849

Compressive 1–5 = Number of scaffolds used for Compressive strength.

### Effect of the powder mixture and production parameters on hardness

As shown in Table 5, the S/N ratios response was computed utilizing the hardness value analysis. The processing parameters were identified and ranked by employing the delta value in Table 5^60^. The value 1 denotes the highest rank. From the table, the sintering temperature has a delta value of 0.61, while compaction pressure and powder mixture have an equal delta value of 0.06. These values are less than the ones obtained by Abifarin^12,14^, but can be attributed to the processing method on the parameters. However, for sintering temperature, compaction pressure, and powder mixture the rankings are 1, 2, and 3, respectively.

**Table 5:** Responses for hardness data Signal to Noise Ratios (Larger is better).

Processing parameters	Sintering temperature (°C)	Compaction pressure (Pa)	Powder mixture (%wt)
Level 1	30.03	30.23	30.30
Level 2	30.12	30.26	30.23
Level 3	30.64	30.30	30.26
Delta	0.61	0.06	0.06
Rank	1	2	3

**Figure 3:**
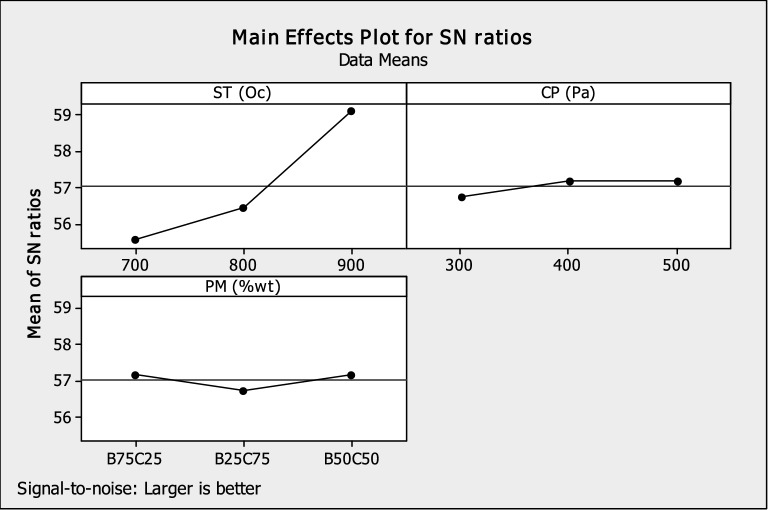
Plots showing the main effects of S/N ratios of hardness.

Figure 3 shows the effect of powder mixture (PM), compaction pressure (CP), and sintering temperature (ST) on HAp hardness value. The plot shapes for sintering temperature, compaction pressure, and powder mixture point demonstrate how changes in these variables have a significant impact on hardness. The plot reveals the following:

- Increasing sintering temperature, compaction load, and powder mixture (more bovine bone) increased SNR's hardness, which is in line with other studies^34,60^. Akpan et al.^11^, stated that bovine bone has more calcium content than catfish bone and claimed that sintering of HAp at 900 °C with a compaction load of 500 Pa would lead to an increase in hardness value.
- While employing a compaction load of 500 Pa with more bovine bone by weight is both safe and economical, raising the sintering temperature lowers the stress created on the pellets' surface during compaction.
- That sintering temperature of 900 °C, compaction pressure of 500 Pa, and powder mixture of B50/C50 is the optimal parameter distribution levels for producing HAp scaffolds with good hardness qualities (see the data presented in Table 5).
- That the hardness S/N ratios significantly decrease at a sintering temperature of 700 °C, compaction pressure of 300 Pa, and powder mixture of B25/C75. This is because, at this temperature, pressure could release the residual tension produced there^34^.

#### Quantitative impact of production factors and powder mixture on HAp hardness

To establish the best parameter levels, an Analysis of Variance (ANOVA) (See Table 6) was done to know the best percentage of contribution (% of C) or the effect of the individual processing parameter effect on the hardness value. The result revealed that:

- The sintering temperature contributing 97.79% is the extreme influencing parameter affecting the HAp hardness value.
- The compaction pressure, powder mixture, and error have insignificant percentage contributions of 0.92, 0.89, and 0.40, respectively. The compaction pressure, powder mixture, and residual error percentages are less than 0.05, hence, they are insignificant on the HAp hardness value^30,48^.
- Also, the values of R^2^ and R^2^_adj_ are very large 99.6% and 98.4% respectively and confirms the validity of this model.

**Table 6:** ANOVA for S/N Ratio of hardness.

Source	DF	Adj SS	Adj MS	F	*p*	Contribution (%)	Remarks
ST	2	0.645151	0.322575	245.68	0.004	97.79	Significant
CP	2	0.006048	0.003024	2.30	0.303	0.92	Insignificant
PM	2	0.005915	0.002957	2.25	0.307	0.89	Insignificant
Error	2	0.002626	0.001313			0.40	Insignificant
Total	8			S = 0.03623		R^2^ = 99.6%	R^2^_adj_ = 98.4%

### Effect of the powder mixture and production parameters on hardness

As shown in Table 7, the S/N ratios response for compressive strength was also computed utilizing the compressive value analysis (Table 4)^61^. From the Table, the sintering temperature has the highest rank, with a delta value of 3.52, which is higher than the figure reported by Abifarin et al.^34^. The compaction pressure and powder mixture both have a rank of 2.5 and a delta value of 0.45.

**Table 7:** Response for compressive data Signal to Noise Ratios (Larger is better).

Processing parameters	Sintering temperature (°C)	Compaction PRESSURE (Pa)	Powder mixture (%wt)
Level 1	55.56	56.74	57.18
Level 2	56.46	57.18	56.74
Level 3	59.08	57.18	57.18
Delta	3.52	0.45	0.45
Rank	1	2.5	2.5

**Figure 4:**
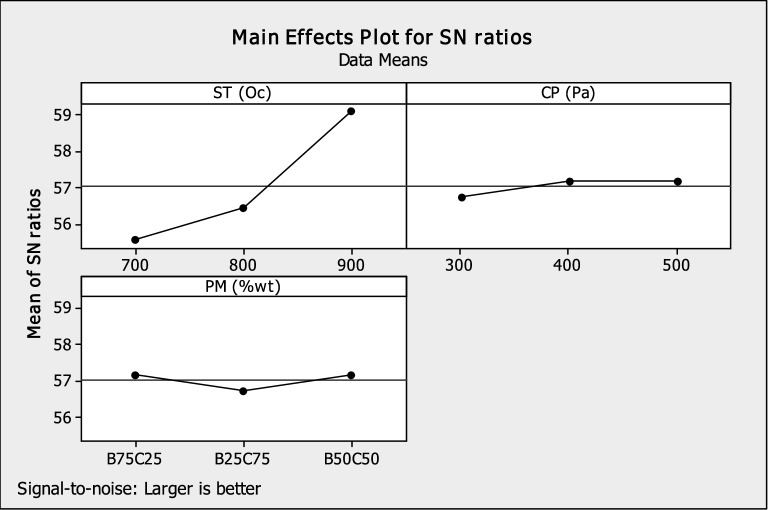
Plots illustrating the main effects of S/N ratios of compressive.

Figure 4 shows the effect of powder mixture (PM), compaction pressure (CP), and sintering temperature (ST) on HAp hardness value. The plot shapes for sintering temperature, compaction pressure, and powder mixture point demonstrate how changes in these variables have a significant impact on the compressive strength. The plot reveals the following:

- That increase in sintering temperature, compaction pressure, and equal HAp powder improved the compressive strength S/N Ratio of HAp scaffolds (i.e. the pellets).
- That higher sintering temperature, compaction pressure and equal powder mixture or greater bovine can be used to produce HAp scaffold with an increased compressive strength (That is, 900 °C, 500 Pa, and B50/C50 or B75/C25 are the expected processing parameter levels for improved compressive strength HAp scaffold).

## Quantitative impact of production factors and powder mixture on HAp compressive strength

An ANOVA was done to establish the percentage contribution of each processing parameter's effect on HAp compressive strength (See Table 8). The result revealed that:

- The sintering temperature is the most influential parameter affecting the compressive strength of the as-produced-HAp scaffold, with a percentage contribution of 94.39%.
- The compaction pressure, powder mixture, and error have insignificant percentage contributions of 1.87.
- Also, the values of R^2^ and R^2^_adj_ are very large 98.1% and 92.5% respectively and the difference is more than 5%. This demonstrates that the model is well-fitting.

**Table 8:** ANOVA for SNR of compressive.

Source	DF	Adj SS	Adj MS	F	*p*	Contribution (%)	Remarks
ST	2	20.11270	10.05640	50.49	0.019	94.39	Significant
CP	2	0.398400	0.199200	1.00	0.500	1.87	Insignificant
PM	2	0.398400	0.199200	1.00	0.500	1.87	Insignificant
Error	2	0.398400	0.199200			1.87	Insignificant
Total	8			S = 0.4463		R^2^ = 98.1%	R^2^_adj_ = 92.5%

### Grey relational analysis (GRA) for the multiple performance characteristics

For the production of a novel mix of HAp scaffolds from biowastes with improved hardness and compressive strength values, it is important to establish the appropriate processing parameter levels. The results of the effect of processing parameters on HAp hardness and compressive strength values revealed that a sintering temperature of 900 °C is necessary to fabricate mechanically improved HAp, but compaction pressure of 500 Pa (0.5 KN) and a powder mixture of B50/C50 or B75/C25 are required for these parameters. Since only one parameter level is to be monitored, it is, therefore, necessary to use grey relational analysis (GRA) to predict the best processing parameters. GRA is most commonly used to solve problems involving a statistically controlled strategy. The experimental mechanical properties of the responses in Tables 3 and 4, in particular hardness and compressive strength, were pre-processed with grey relational generation. The data were normalized using Eq. (2). The deviation sequences were then computed using Eq. (3). The reference and deviation sequences obtained following data pre-processing are shown in Table 9. The Grey Relational Coefficient (GRC) was then calculated using Eq. (4). The grey relational grade (GRG) was calculated using the average of the GRCs. The corresponding S/N Ratios were calculated from the GRGs, as shown in Table 10. A greater S/N ratio percentage value is acceptable and confirms that the investigated mechanical parameters are close to desired normalized GRG value^62^.

**Table 9:** Reference and deviation sequence after pre-processing of data.

Experimental run	Reference sequence, x*i		Deviation sequence, ${\Delta }_{oi}$	
	Mean, hardness	Mean, compressive strength	Mean, hardness	Mean, compressive strength
1	0	0	1	1
2	0.142857	0.166667	0.958333	0.833333
3	0.285714	0.25	0.958333	0.75
4	0.428571	0.166667	0.958333	0.833333
5	0.571429	0.5	0.875	0.5
6	0.714286	0.333333	0.708333	0.666667
7	0.857143	0.75	0.041667	0.25
8	1	0.875	0	0.125
9	1.142857	1	0.041667	0

**Table 10:** Rank of grey relational grade (GRG) with S/N Ratio.

Exp. run	Mean, hardness	Grey relational coefficient, $\varepsilon i\left(k\right)$			
		Mean, compressive strength	GRG, $\gamma i$	S/N Ratio of GRG	Rank
1	0.333333	0.333333	0.333333	− 9.54243	9
2	0.342857	0.375	0.358929	− 8.89984	8
3	0.342857	0.375	0.358929	− 8.89984	8
4	0.342857	0.375	0.358929	− 8.89984	8
5	0.363636	0.428571	0.396104	− 8.04382	5
6	0.413793	0.428571	0.421182	− 7.5106	4
7	0.923077	0.428571	0.675824	− 3.40333	3
8	1	0.5	0.75	− 2.49877	2
9	0.923077	1	0.961538	− 0.34067	1

**Figure 5:**
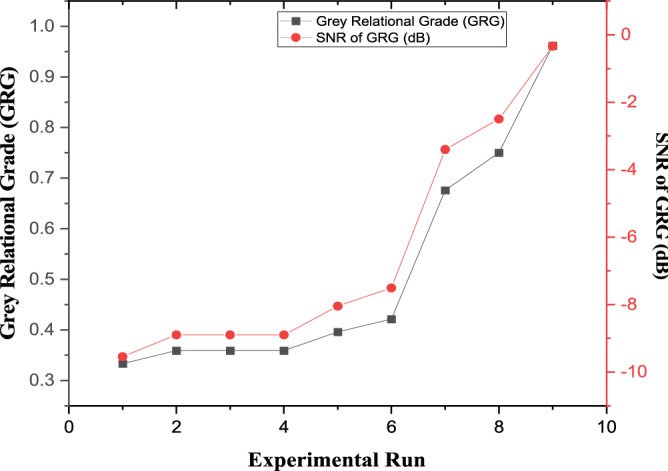
Relationship between GRG and S/N Ratios.

From Table 10, experimental run 1 has the highest significant S/N Ratio. Similarly, the major rank was assigned to experimental run 9. Figure 5 depicts the relationship between GRG and S/N ratios, which attest to the significance of the GRG^34^. The GRG of the relevant factor at the specific level was selected, and an average value was approximated to establish the GRG mean for specific elements. In experimental runs 7, 8, and 9, the processing parameter (sintering temperature), was set to level 1. For the calculation, the corresponding GRG values from Table 10 were employed, as given in Eq. (5).

Following the steps indicated above, mean of the selected GRGs was calculated (See Table 11). It was discovered to be 0.5128, which is close to the value found in Ref^34^. The percentage of the link between the reference sequences and the GRA correspondence sequence is represented by the grades. A good relationship is indicated by higher GRG mean values^62^. From Table 11, it is possible to predict the best processing parameters for the production a novel mix HAp scaffold from biowastes. Sintering temperature at level 3, compaction pressure at level 3, and powder mixture at level 2 are the peak grey relational grades in Table 11. As a result, the sintering temperature of 900 °C, the compaction pressure of 500 Pa, and the powder mixture of B50/C50 are the ideal processing parameters for the production of a mechanically enhanced novel mix HAp scaffold from biowastes. As confirmed by Abifarin et al.^34^, an increase in sintering temperature improves the mechanical property of HAp.

$$Sintering \,Temperature\, \left( {level 3} \right) = \frac{0.6758 + 0.7500 + 0.9615 }{3} = 0.7958$$

**Table 11:** Response Table for Means (Larger is better).

Processing parameters	Sintering Temperature (^o^C)	Compaction Pressure (Pa)	Powder Mixture (%wt)
Level 1	0.3504	0.4560	0.5015
Level 2	0.3921	0.5017	0.5598
Level 3	0.7958	0.5805	0.4770
Delta	0.4454	0.1245	0.0828
Rank	1	2	3

Mean of GRG = 0.5128.

### Analysis of variance (ANOVA) for grey relational grade (GRG)

An ANOVA was performed for the grey relational grade at a 95% confidence level to determine the relevance and percentage contribution of each processing parameter on the novel mix of HAp scaffolds. Based on the two responses (hardness and compressive strength), Table 12 revealed that:

- The sintering temperature (ST) has the highest notable influence on the GRG (88.68%).
- The compaction pressure (CP) is next, with a contribution of 5.82%.
- The powder mixture (PM) and residual error, with contributions of 2.65% and 2.85%, respectively, are insignificant.
- The high R values indicate that the constructed model is well-fitting.

**Table 12:** ANOVA for Grey Relational Grade.

Source	DF	Adj SS	Adj MS	F	P	Contribution (%)	Remarks
ST	2	0.36310	0.181548	31.07	0.031	88.68	Significant
CP	2	0.02381	0.011905	2.04	0.329	5.82	Significant
PM	2	0.01086	0.005432	0.93	0.518	2.65	Insignificant
Error	2	0.01169	0.005843			2.85	Insignificant
Total	8			S = 0.07644		R^2^ = 97.1%	R^2^_adj_ = 88.6%

### Confirmation analysis

After observing the optimal processing parameter levels for the production of the novel mix HAp scaffold, the response was predicted as given in Eq. (6)^33,34^:

Hence, from Eq. (6) and Table 11;

$$\begin{aligned} \gamma_{predicted} = &\, 0.5128 + \left( {0.7958 - 0.5128} \right) + \left( {0.5805 - 0.5128} \right) + \left( {0.5598 - 0.5128} \right) \\ \gamma_{predicted} = & \,0.5128 + 0.2830 + 0.0677 + 0.0470 = 0.9105 \\ \end{aligned}$$

Also,

$$\gamma_{Experimental} = \frac{0.6758 + 0.7500 + 0.9615 }{3} = 0.7958$$

From the calculations, it is clear that the predicted GRG (0.9105) is greater than the experimental GRG (0.7958).

Applying Eq. (8), Confidence Interval (CI) was calculated as follows:

Thus:

$$Confidence\, Interval = \sqrt {F_{\alpha } \left( {1,f_{e} } \right)V_{e} \left[ {\frac{1}{{n_{eff} }} + \frac{1}{X}} \right]}$$

*F*_∝_(1, *fe*) = F ratio essential for α = *F*_∝_(1, 2) = 18.51 (From F tables); α = risk; *f*_*e*_ = DOF of error (DF) = 2; V_e_ = variance of error = 0.005843; η_*eff*_ = effective number of repetitions = 1.3, which is the Eq. (9):

$$n_{eff} = \frac{S}{{1 + \left( {Total \,DOF\, of \,control\, factors} \right)}}$$

X = number of repetitions for trial confirmation = 1; S = Sum of trials = 9 and Total DOF of control factors = 6

$$\therefore n_{eff} = \frac{9}{1 + 6} = \frac{9}{7} = 1.3$$

Hence, $$\mathbf{CI } = \sqrt {18.51 \times 0.005843\left[ {\frac{1}{1.3} + \frac{1}{1}} \right]}{ = }\sqrt {18.51 \times 0.005843\left[ {\frac{1}{1.3} + \frac{1}{1}} \right]} = \pm 0.4374$$.

According to Abifarin^12^: $\gamma_{predicted} - CI <$ $\gamma_{Experimental}$ ˂ $\gamma_{predicted} + CI$ i.e. 0.4374 ˂ 0.7958 ˂ 1.3479.

The experimental grey relational grade was 0.7958, which is within the 95% confidence interval of the projected optimal grey relational grade^34^. The confidence interval shows that this value is close to the projected ideal grey relational grade, indicating that the optimal processing parameter levels are effective in producing an improved mechanically novel mix HAp scaffold from biowastes (bovine and catfish bones).

### Characterization of the optimum sample

#### SEM/EDX results

**Figure 6:**
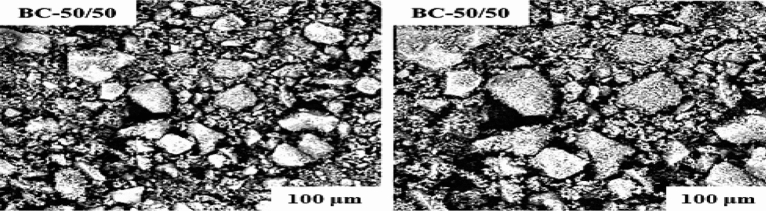
SEM result of the optimum parameter.

Figure 6 displays the surface morphology of the optimum parameter (B50/C50) at low magnifications of (300 × and 500 ×. On nanometric scales, aggregations can be observed. The image shows big, gritty grains that are not tightly packed. Presumably, these big grains include embedded porosities. To know the samples' compositional makeup, SEM–EDX analysis was done. Table 13 shows that the EDX results are consistent with those earlier published^14^. The sample contained calcium (Ca), phosphorous (P), and oxygen (O), with minor elements such as magnesium (Mg), strontium (Sr), and potassium (K) also present. In in vitro and in vivo analysis, the attendance of these components can increase and speed up bone growth and new bone creation. The presence of Mg in particular is critical for bone metabolism and the creation of artificial bones^61^. Using a simple Microsoft Excel software, from Table 13, the Ca/P ratio of 2.86 was found for the optimum parameter. One explanation for this variation is the presence of a foreign crystal, which might be any of the calcium-rich compounds CaO, Ca(OH), CaCO_3_, or a combination of the three. When employed as scaffolding materials for bone remodeling, a high Ca/P ratio has been recommended to assure the HAp's best biocompatibility and chemical stability in the implanted area^63^.

**Table 13:** EDX result of the produced hydroxyapatite optimum parameter (B50/C50) at 900 °C and 500 Pa.

Elements	Sample (B50/C50)								
	O	Mg	Al	Si	P	K	Ca	Fe	Sr
Concentration (wt%)	37.041	1.154	1.119	0.884	12.153	0.073	45.503	0.313	0.118

#### XRD result

**Figure 7:**
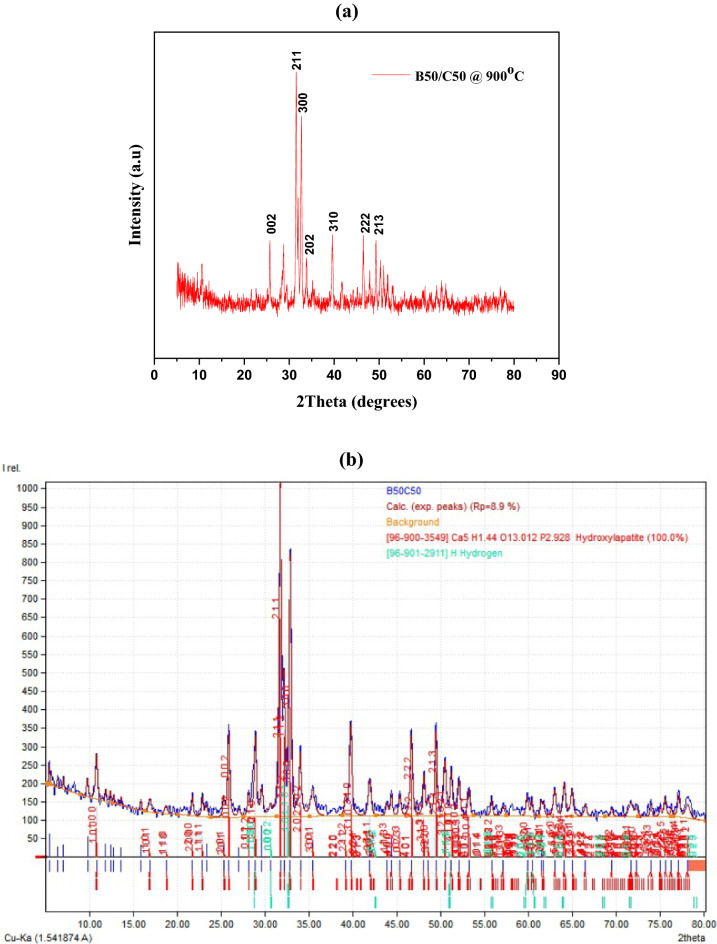
XRD result of the optimum sample (**a**) Origin Pro plot (**b**) Match analysis.

The phase and purity of the resultant powders were identified using XRD analysis. All of the samples' reflections were in good agreement with the ICDD standard card files for HAp (JCPDS File No. 09-0432), which suggests that pure phase HAp was formed (See Fig. 7). The B50/C50 had a higher degree of crystallinity and mean crystallite size of 80.42% and 27.3 nm, respectively. A higher crystallinity shows crystallite development and a decrease in the system's free energy. Notably, as the reaction kinetics depends on the surface area, crystallite sizes are crucial for applications involving bone regeneration. More reactivity is present when the particle size is smaller. The sample has a porosity of 49.02% and an apparent density of 1.61 g/cm^3^ which is within the permitted range for HAp. Porosities ranging from 40 to 90% have been shown to improve osteointegration^14,64^. The calculated porosities confirmed that the matrix of the sample is suitable for biomedical purposes. It is important to note that B50/C50 (hydroxyapatite) has a hexagonal shape and space group of p63/m.

## Conclusion

The quantitative effect of the powder mixture, compaction pressure, and sintering temperature has been studied with the help of the Taguchi orthogonal array technique assisted by grey relational analysis (GRA).

The result revealed an inconsistency in the powder mixture as the optimum state for individual mechanical properties, but the grey relational analysis (GRA) showed better mechanical properties with a powder mix of B50/C50, 500 Pa compaction pressure, and 900 °C sintering temperature as the best production parameters for the fabrication of mechanically enhanced novel mix HAp from biowastes for biomedical use. The obtained result further showed that the novel mix of these powders is a good and promising material for high-strength biomedical applications, having a contribution of 97.79% on hardness and 94.39% on compressive strength of HAp. The obtained experimental grey relational grade of 0.7958 is within the 95% confidence interval, according to confirmation analysis (CA). These results are expedient in the field of biomedical engineering for the production of a novel mix of HAp powder from other natural sources for the orthopedics industry to fabricate a mechanically fitted HAp for load-bearing clinical application. The sample had a higher degree of crystallinity and mean crystallite size of 80.42% and 27.3 nm, respectively. The SEM images showed big, gritty grains that are not tightly packed.

## Data Availability

All data generated or analyzed during this study are included in this published article.
